# The Morphology of the Genital Tubercle: A Macroscopic Assessment and Ultrasound Approach on Early Fetal Sex Determination

**DOI:** 10.3390/diagnostics16162626

**Published:** 2026-08-19

**Authors:** Octavian Munteanu, Monica-Mihaela Cirstoiu, Roxana Bohiltea, Andra-Ioana Baloiu, Maria Voinea, Razvan-Adrian Covache-Busuioc, Matei Serban, Adrian Tulin, Iulian Slavu, Zoran Filipoiu, Ioan-Andrei Petrescu, Ciprian Coroleuca

**Affiliations:** 1 Department of Anatomy, “Carol Davila” University of Medicine and Pharmacy, 050474 Bucharest, Romania; 2 Department of Obstetrics and Gynecology, Faculty of Medicine, “Carol Davila” University of Medicine and Pharmacy, 050474 Bucharest, Romania; 3 Department of Obstetrics and Gynecology, Faculty of Stomatology, “Carol Davila” University of Medicine and Pharmacy, 050474 Bucharest, Romania; 4 Faculty of Medicine, “Carol Davila” University of Medicine and Pharmacy, 050474 Bucharest, Romania; 5 Puls Med Association, 051885 Bucharest, Romania

**Keywords:** genital tubercle, morphology, sonography, fetal sex determination, first-trimester ultrasound, 3D ultrasonography, embryology, external genitalia

## Abstract

**Background/Objectives:** The genital tubercle develops in the fourth week from mesenchymal cells in the lateral edges of the embryo. In females the genital tubercle develops into the clitoris while in males it participates in forming the penis. The aim of this study was to perform a macroscopic as well as an ultrasonographic assessment of the genital tubercle and its gender-dependent derivates, presenting a parallel comparative evaluation between the two. **Methods:** We performed a morphological assessment of the genital tubercle through micro-dissection of 141 embryos and fetuses with gestational ages between 5 and 14 weeks, in the Department of Anatomy of “Carol Davila” University of Medicine and Pharmacy. An ultrasound imaging study of the genital tubercle using 3-dimensional ultrasonography (3DUS) was also performed in the Department of Obstetrics and Gynecology of University Emergency Hospital of Bucharest. The morphology of the genital tubercle was analyzed by ultrasound scanning of 119 embryos and fetuses with gestational ages between 5 and 14 weeks. The age of all the embryos was determined by measuring the crown–rump length. **Results:** The dimensions and especially the direction of the genital tubercle were evaluated and compared in order to establish whether sex determination is possible as early as 7–8 weeks of gestation. The genital tubercle orientates cranially in males and caudally in females as early as 6 weeks of gestation, during the bi-potential phase. **Conclusions:** The orientation of the genital tubercle plays an essential role in establishing the sex of the fetus, regardless of its size.

## 1. Introduction

The male and female reproductive systems derive from the same embryonic tissue; therefore, the development of genitalia involves transitioning through an indifferent stage, also known as a bi-potential phase, in which the gonads, genital ducts and external features will initially be alike in both sexes. As a result, the fetus has bi-potential sexual development for the first 3 months of life [1].

The phenotype depends on the presence of sex chromosomes and the biochemical and hormonal milieu. However, sex is established at the time of fecundation by the nature of the chromosomal composition of the spermatozoon, which contains either a Y chromosome which has a dominant effect (23, Y constitution), or an X chromosome (23, X constitution) [2].

The genital tubercle develops in the fourth week from mesenchymal cells in the lateral edges of the embryo. The urethral groove, surrounded by the primary urethral folds, and the anal pit appear on the midline. The labio-scrotal swellings develop lateral to the urethral folds. Three elements underlie the development of the external genitalia: two urogenital folds, the genital tubercle and a pair of labio-scrotal folds [3].

In females, the absence of androgen induces specific transformations: the genital tubercle develops into the clitoris. The urogenital orifice becomes the vulvar cleft, while the genital folds enlarge and overgrow the genital tubercle becoming the labia minora. Simultaneously, the labio-scrotal folds will form the labia majora [4].

In males, the genital tubercle begins to elongate in the 10th week. The genital folds fuse and form the shaft and glans of the penis. The fusion encloses the definitive urogenital sinus, forming the penile urethra. An invagination of ectoderm covering the glans penis forms the distal most urethra, while the scrotum is formed from the labio-scrotal folds, also known as the genital folds [5].

In 2-dimensional ultrasonography (2DUS), the cranial direction of the genital tubercle for males and caudal for females, associated with the presence of a cranial notch in males and a caudal notch in females, named the sagittal sign, can predict the fetal sex in the late first and early second trimesters (usually after 11 weeks of gestation) [6].

During the second and third trimesters of pregnancy, the cardinal elements on which the determination of fetal sex is based are the presence and the size of the penis in males or labial folds in females. When performing transvaginal ultrasound, male sex determination is based on the visualization of three elements, respectively: the fetal scrotum, the cranially directed phallus and the longitudinal raphe at the base of the penis; in female fetuses, the diagnostic criteria consists of the presence of two or four parallel lines, which represent the labial folds, and the caudally directed phallus (clitoris) [7,8].

The aim of this study was to perform a macroscopic as well as an ultrasonographic assessment of the genital tubercle and its sex-dependent derivates, presenting a parallel comparative evaluation between the two.

## 2. Materials and Methods

This study was performed between 1st November 2023 and 15th February 2026, consisting of a morphological assessment of the genital tubercle on formalin fixed human embryologic and fetal specimens, as well as an ultrasonographic in utero evaluation of embryos and fetuses.

We performed a morphological assessment of the genital tubercle through micro-dissection of 150 embryos and fetuses with gestational ages between 5 and 14 weeks, in the Department of Anatomy of “Carol Davila” University of Medicine and Pharmacy. The gestational ages have been determined by measuring the crown–rump length of the subjects.

All specimens had been previously fixed in 10% neutral-buffered formalin solution for 3 days. The specimens were extracted from the formaldehyde solution and rested on a surgical tray to dry, thus assuring the differentiation of the small-scale features that could otherwise be deteriorated by manual drying attempts. The sex of the aborted fetuses was confirmed after a complete necropsy, by analyzing the morphological features of the internal genitalia (we analyzed the dimensions, direction and orientation of the Müllerian ducts, the location of the gonads, and performed microscopic evaluation of the gonads, when necessary; e.g., female sex in embryos with fused Müllerian ducts forming the future fallopian tubes and utero-vaginal canal). In 14 cases, genetic testing has been performed in order to confirm the sex of the embryo.

Specimens which presented substantial damage in the pelvic region (*n* = 9) due to either obstetrical procedure or preparation circumstances were excluded from further analysis. Therefore, 141 specimens have been included in the morphological branch of this study.

An ultrasound imaging study of the genital tubercle using 3-dimensional ultrasonography (3DUS) was also performed in the Department of Obstetrics and Gynecology of University Emergency Hospital in Bucharest. The morphology of the genital tubercle was analyzed by ultrasound scanning of 150 embryos and fetuses with gestational ages between 5 and 14 weeks. Gestational age was calculated from crown–rump length in conjunction with menstrual or ovulation dating.

The ultrasound examinations were performed either transvaginally or transabdominally using a high-resolution ultrasound system equipped with both 2D and 3D capabilities, specifically the Voluson E8 Expert (General Electric Company, Wauwatosa, WI, USA), 54 Hz, software version 13, 3D volume acquisition. Ultrasonographic acquisition was performed using either transvaginal or transabdominal approach as determined by the position of the gestational product, and all scans were obtained and assessed by three highly experienced examiners (O.M., M.-M.C., R.B.). The fetus was assessed in a true midsagittal plane, obtained according to standard first-trimester anatomical landmarks: visualization of the profile including the forehead, nasal bone, palate, chin, spine, and lumbosacral region in a single continuous plane. The direction of the genital tubercle was evaluated in the midsagittal plane of the fetus, by assessing the direction of the genital tubercle in this plane, i.e., the sagittal sign. The genital tubercle angle (GTA), a method commonly used for several years, was also determined by imaging the midsagittal plane of the fetus, by measuring the angle between a line drawn along the lumbosacral spine of the fetus, and another one from the genital tubercle. The fetus is assigned male sex if the angle is >30°, and female sex if the genital tubercle is parallel or convergent (<10°) to the horizontal line. At an intermediate angle of 10–30° the sex is not determined, and a follow-up ultrasound was performed after 7–10 days. To enhance reproducibility, only true midsagittal images with a neutral fetal position and clear visualization of the tubercle (separate from the umbilical cord) were accepted. Measurements were performed on magnified still frames. However, due to suboptimal positioning or umbilical cord interference, the examiners could establish the sex of the embryo in only 119 cases.

One hundred randomly selected images were observed again blindly by the same investigator one month after the first round of observations. Examiner consistency was assessed for all variables. Variables were highly similar between the first and second rounds of observations, with correlation coefficients of 0.84–0.97. The sex of the fetus was reconfirmed later in the course of the pregnancy, at 20 weeks. When available, we consulted birth records and postpartum neonatal charts.

The dimensions and especially the direction of the genital tubercle were evaluated and compared in order to establish whether sex determination is possible as early as 7–8 weeks of gestation.

The procedures and tests we conducted adhere to the Helsinki Declaration of 1975, as amended in 2000, as well as in the national legislation. Written consent from all patients who consented to have their aborted fetuses utilized in the study was gathered. The Ethical Committee of the “Carol Davila” University of Medicine and Pharmacy has approved the study (approval no. 31760; 24 October 2023).

## 3. Results

### 3.1. The Indifferent Stage

**Figure 1:**
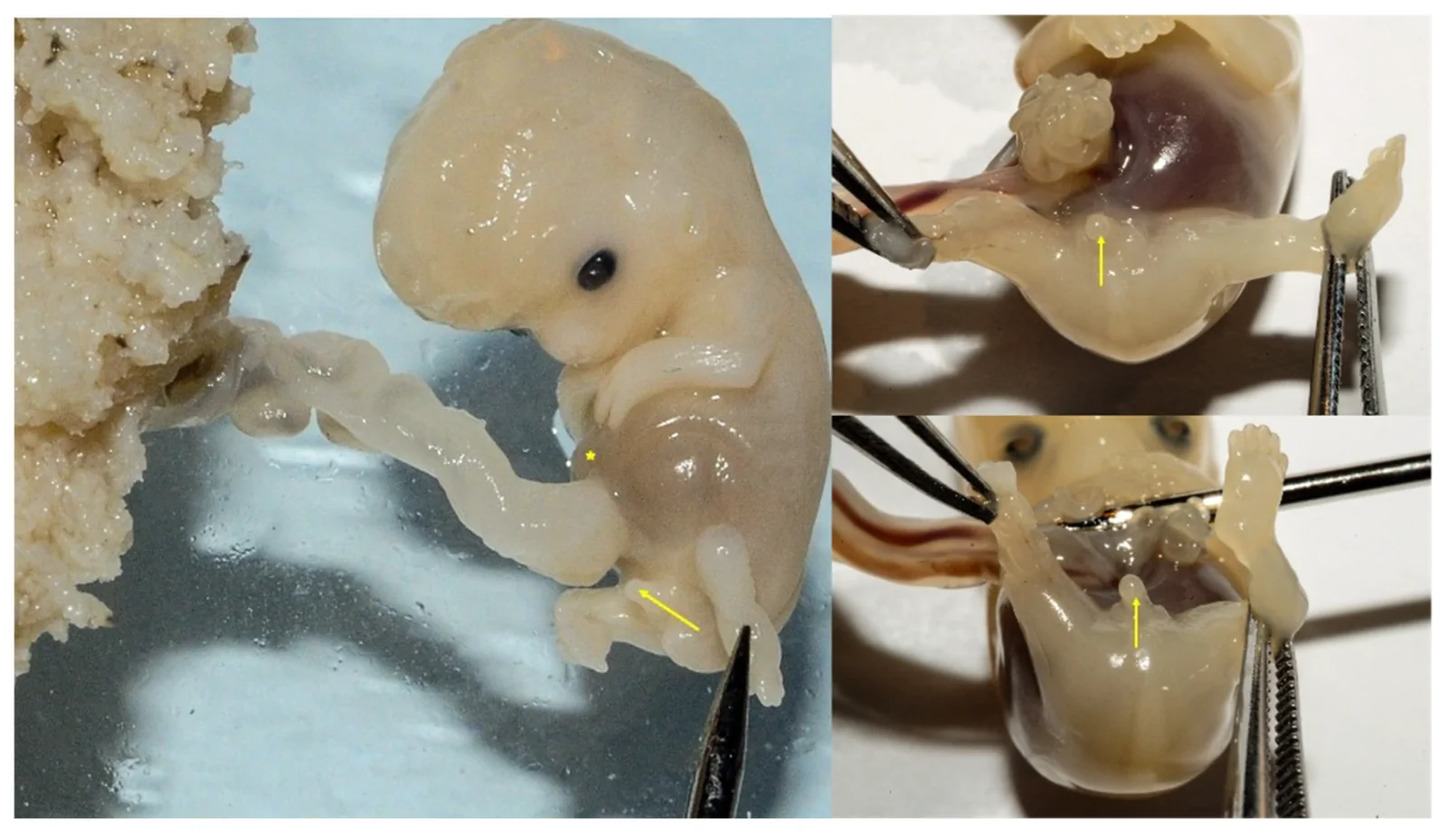
External aspect of the perineal region of a 6-week male embryo. Note the physiological herniation (yellow asterisk) which begins in the 6th week of development and ends in the 8th week. Observe the orientation of the genital tubercle (arrow)—it is directed cranially, facilitating the approach and further the fusion of the labio-scrotal folds on the midline.

**Figure 2:**
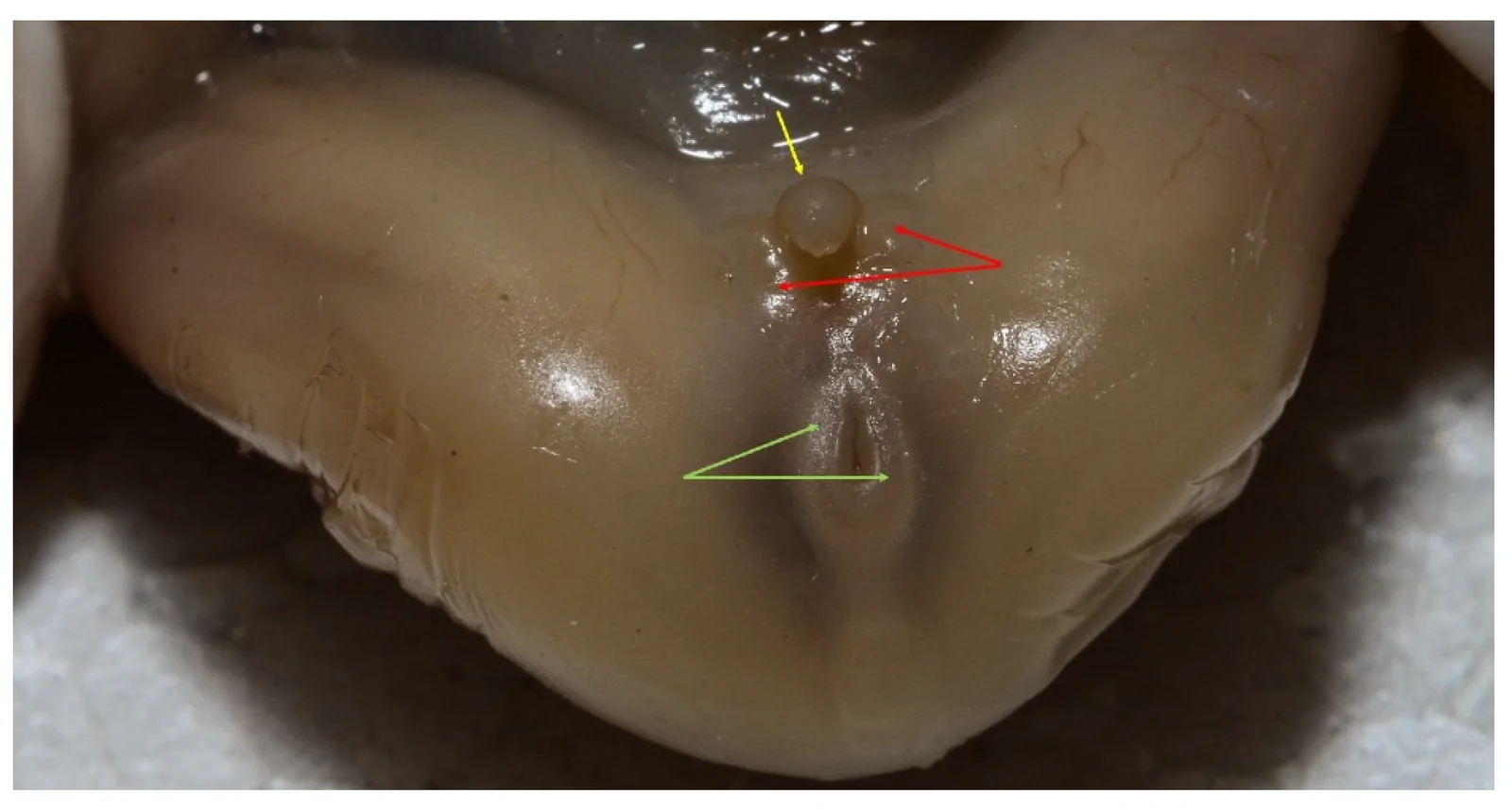
Inferior view of anogenital region of a 6–7 week female individual. Yellow arrow—genital tubercle oriented caudally. Red arrows—labio-scrotal folds. Green arrows—anal folds which result from focal depressions along the cloacal membrane.

The indifferent stage in the development of the genitalia lasts until around the eighth week of gestation, when sexual differentiation begins. During this stage, both male and female embryos present a series of identical features: the genital tubercle and the two labio-scrotal swellings evident on either side of the genital tubercle, around its base (see Figure 1 for a male embryo of 6 weeks, and Figure 2 for a female embryo of the same gestational age). Nonetheless, sex can be established by analyzing the orientation of the genital tubercle—a cranially oriented tubercle suggests male sex, while a caudally oriented one is consistent with female sex.

### 3.2. The Evolution of the Genital Tubercle in Male Embryos

In male individuals, the genital tubercle begins to orient cranially since the initial stages of development, as early as 5 to 6 weeks of gestation, forming an acute angle with the lower anterior wall of the abdomen (see Figure 1).

**Figure 3:**
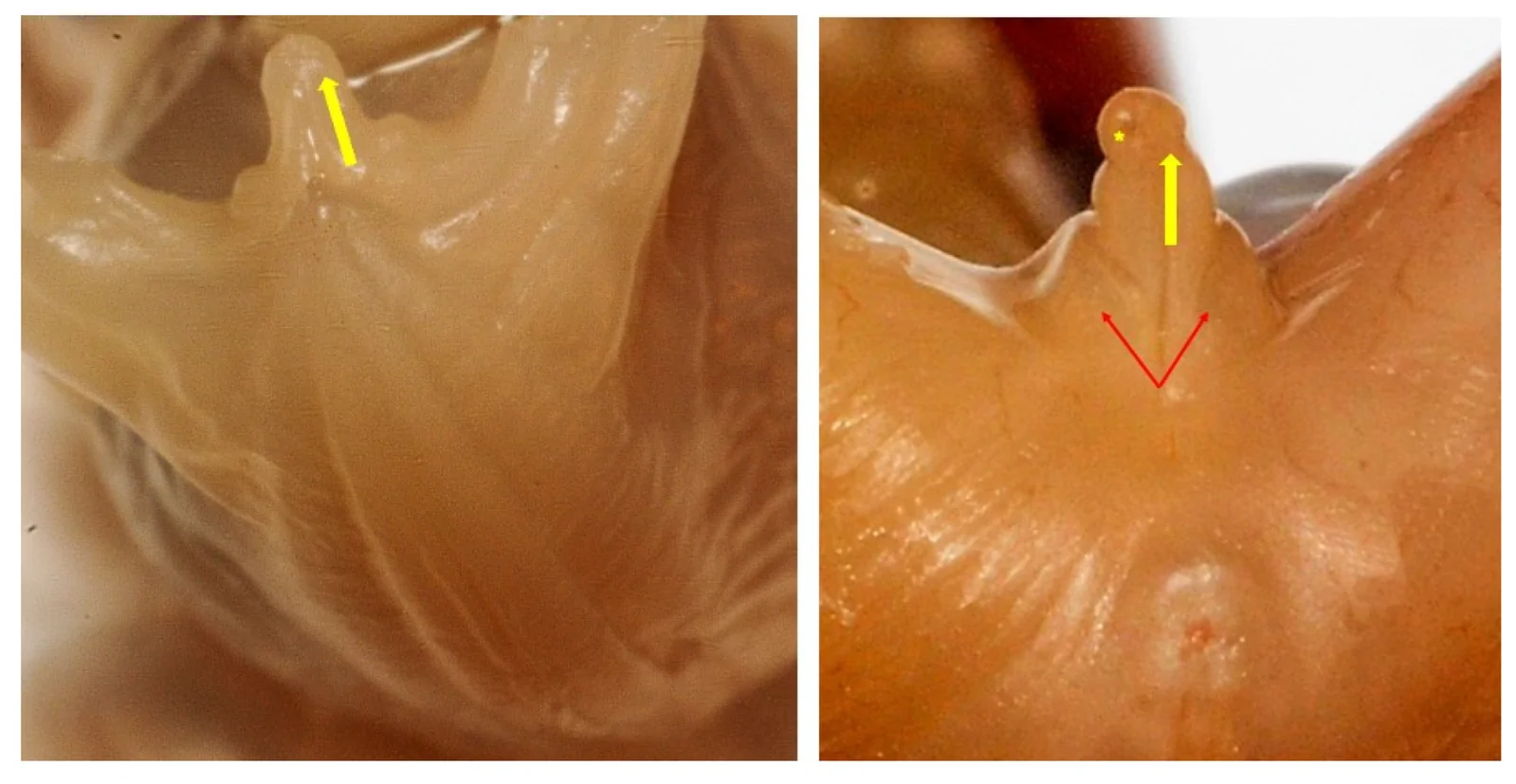
External aspect of the perineal region of a 5–6-week male embryo. Genital tubercle (yellow arrow) is oriented cranially. Labio-scrotal folds (red arrow) begin to fuse towards the midline. Note the urethral notch (asterisk).

Other features that can be distinguished in an embryo of 5–6 weeks of gestation, besides the physiological hernia present at this stage in the development (see Figure 1), are the labio-scrotal swellings—paired dome-shaped structures which can be visualized near the base of the genital tubercle, on either side (see Figure 3).

In male embryos of 6–7 weeks of gestation, the genital tubercle starts to enlarge, maintaining the cranial orientation. The urethral groove becomes evident on the ventral aspect of the genital tubercle, as a median longitudinal depression bordered laterally by two urethral folds. The labio-scrotal swellings have yet to fuse at this stage (see Figure 3).

Between 7 and 8 weeks of gestation, the urethral folds fuse on the midline; consequently, the urethral groove will start to enclose anteriorly starting from the base of the genital tubercle, forming the penile urethra (see Figure 3). The penile anterior raphe represents the line along which the two borders of the urethral groove fuse anteriorly.

This orientation of the phallus also facilitates the inferio-medial migration and midline fusion of the genital folds which form the midline scrotal raphe which is continuous with the penile raphe anteriorly (see Figure 3). Synchronously, the coronal sulcus appears on the anterior aspect of the genital tubercle, as a transverse indentation extending across the distal extremity of the genital tubercle, delimiting the glans.

**Figure 4:**
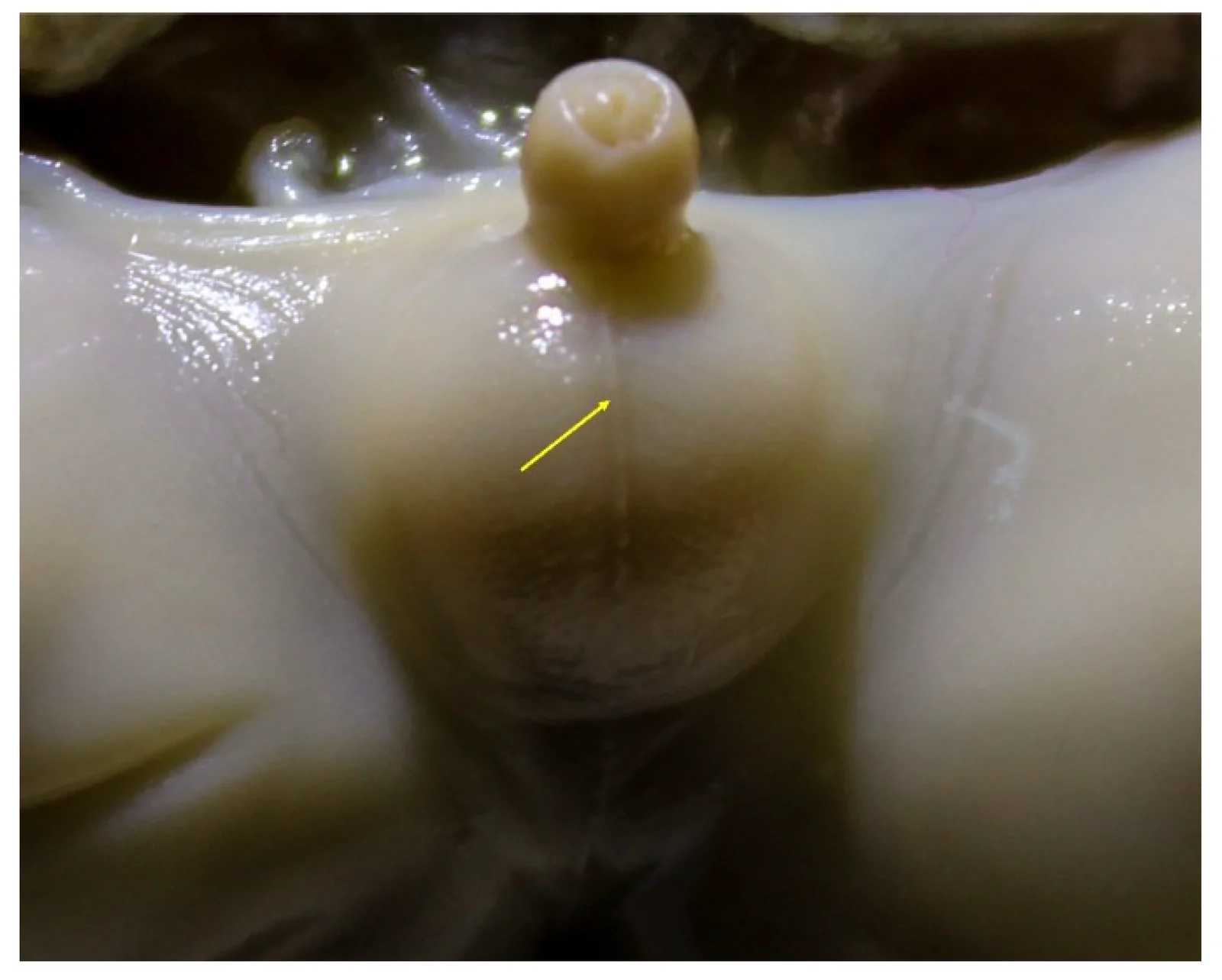
External view of the perineal region of a 10–11-week male fetus—the two labio-scrotal folds are fused on the median line, causing the appearance of the scrotal raphe (yellow arrow).

Upon examining male fetuses of 10 weeks of gestation, one can observe the cranial direction of the genital tubercle, the penile raphe distinguishable on the anterior surface of the penis which is continuous from the base of the penis posteriorly with the scrotal raphe, separating the two hemiscrotums (see Figure 4).

### 3.3. The Evolution of the Genital Tubercle in Female Individuals

**Figure 5:**
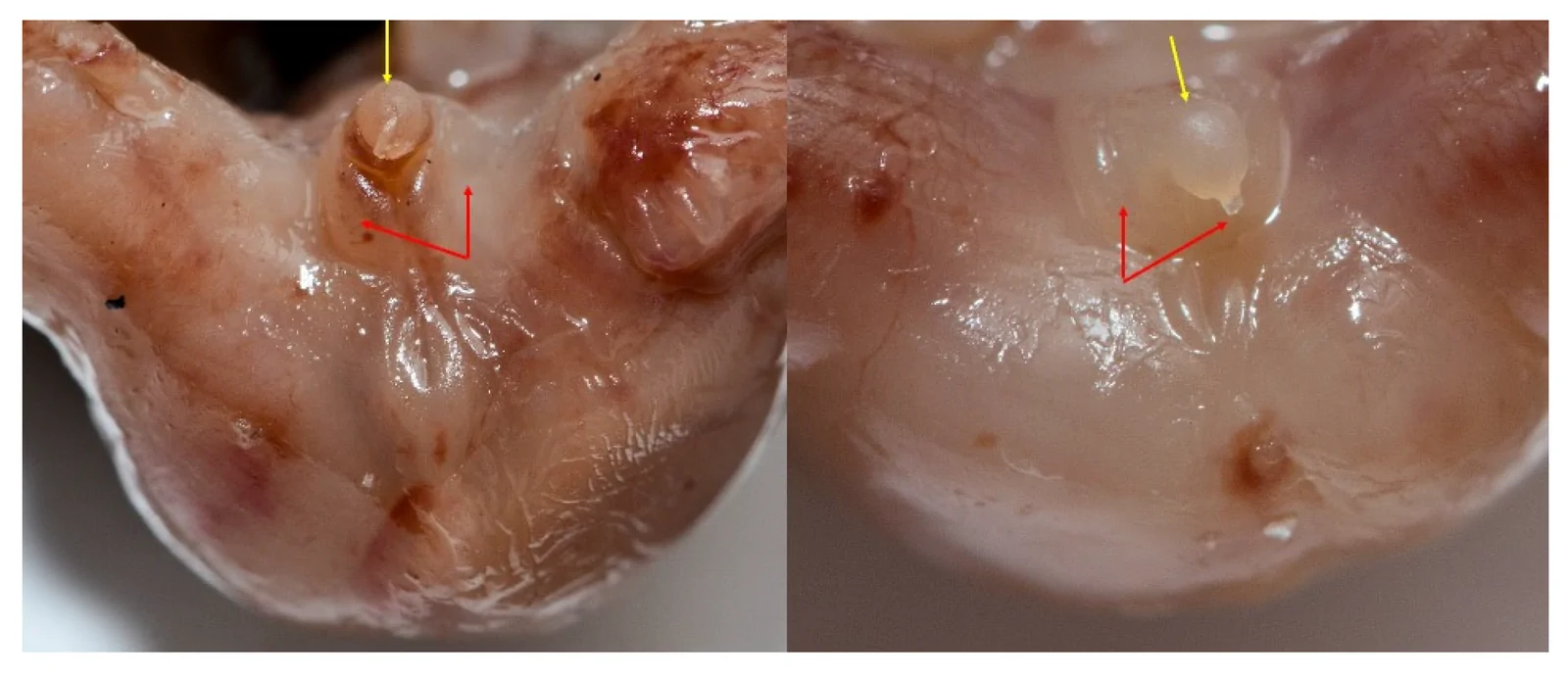
External view of the perineal region of a 6–7-week female fetus. Note the genital tubercle (yellow arrow) is oriented caudally. Laterally, labio-scrotal folds (red arrows) will further form labia majora.

In female embryos, the diagnostic criteria for female sex were evident as early as 6 weeks of development; as of this stage, the genital tubercle is oriented caudally, presenting a caudal notch (see Figure 5).

At approximately 6–7 weeks of gestation, the labio-scrotal swellings are recognizable on either side of the genital tubercle, around its base. The phallus, which will form the future clitoris, aims downward and interposes between the two labio-scrotal swellings, preventing their fusion on the median line. At this stage in the development, the anal folds are also discernible extending sagittally posterior to the genital tubercle (see Figure 2).

As the embryo continues to develop, around 7–8 weeks of gestation, the genital tubercle extends caudally, further isolating the labio-scrotal swellings, which will give rise to the labia majora. Concomitantly, the anal folds continue to develop (see Figure 2).

**Figure 6:**
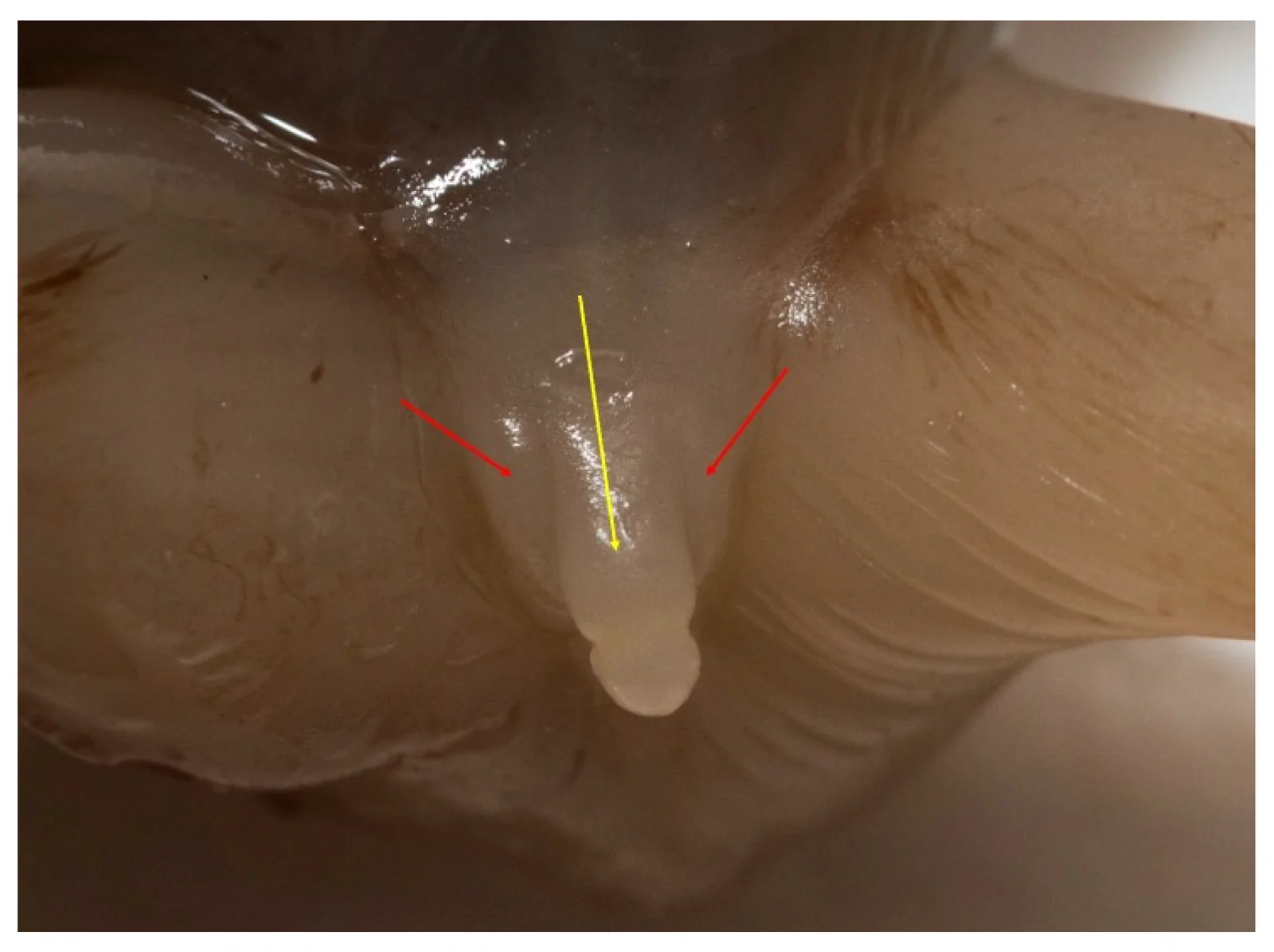
Anterior view anogenital region of a 7–8-week female individual. Genital tubercle (yellow arrow) appears abnormally large for an expectedly female specimen; yet, orientation of the genital tubercle guides the sex of the fetus. Red arrows—unfused labio-scrotal folds (labia majora primordia).

**Figure 7:**
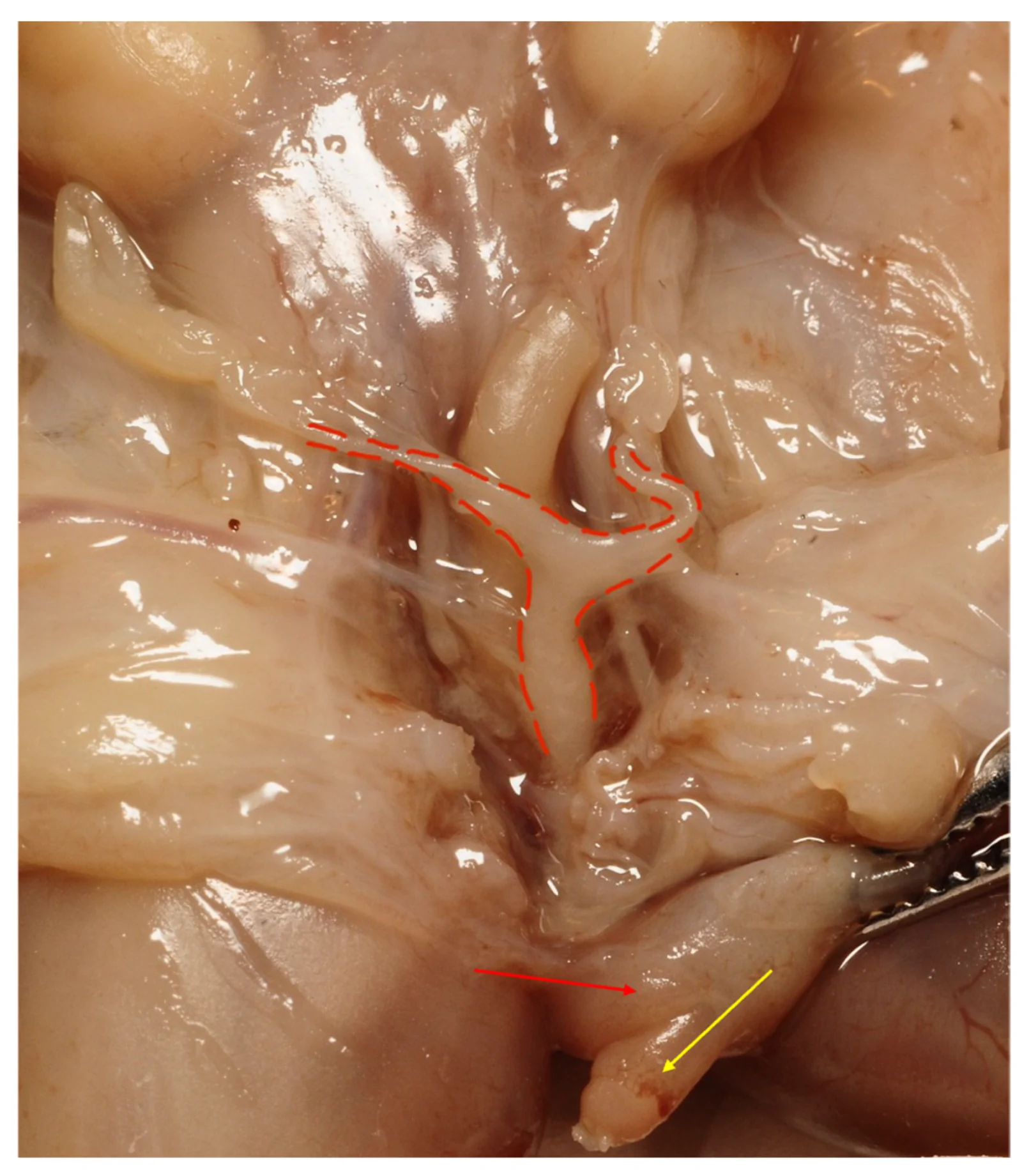
Dissection of the pelvic space in the 7–8-week female embryo. Yellow arrow—genital tubercle, oriented caudally. Red arrow—right labio-scrotal fold. Red dotted line—paramesonephric ducts fused together inferiorly, forming the primordia of the fallopian tubes and the utero-vaginal canal.

One of the specimens presented an enlarged genital tubercle, suggestive of the male sex. Nevertheless, the genital tubercle pointed caudally, separating the two labio-scrotal swellings (see Figure 6). Upon dissection, the presence of the paramesonephric ducts of Muller—the primordium of the uterus and fallopian tubes—certified that the specimen is a female (see Figure 7).

As shown in Figure 6, the external appearance of the fetal genital organs, based on the dimension of the phallus, suggests male sex. During the dissection of the fetal pelvis, we were able to observe the presence of the fused Müllerian ducts forming the future fallopian tubes and utero-vaginal canal (see Figure 7).

### 3.4. Statistical Analysis in the Morphological Branch of the Study

The 141 embryos and fetuses included in the morphological branch of the study have been categorized in three groups, depending on the gestational age of the specimens, as follows: 5 to 8 weeks of gestation (*n* = 29, 16 males and 13 females); 9 to 11 weeks of gestation (*n* = 44, 19 males and 25 females); and 12 to 14 weeks of gestation (*n* = 68, 36 males and 32 females). By analyzing the orientation of the genital tubercle in the first group, and the aspect of the external genitalia in the last two groups, across gestational age groups, diagnostic performance improved with increasing weeks. The distribution of the specimens and assessment of sex are summarized in Table 1. Diagnostic performance was further evaluated by calculating sensitivity, specificity, positive predictive value (PPV), negative predictive value (NPV), and overall accuracy. Male sex was considered the positive outcome. Sensitivity was defined as the proportion of male embryos or fetuses correctly identified by the diagnostic method and was calculated as [correct males/(correct males + incorrect males)]. Specificity was defined as the proportion of female embryos or fetuses correctly identified and was calculated as [correct females/(correct females + incorrect females)]. The positive predictive value (PPV) represented the probability that a specimen classified as male was truly male and was calculated as [correct males/(correct males + incorrect females)]. The negative predictive value (NPV) represented the probability that a specimen classified as female was truly female and was calculated as [correct females/(correct females + incorrect males)]. Overall accuracy represented the proportion of correctly classified specimens among all examined embryos and fetuses and was calculated as [(correct males + correct females)/(correct males + correct females + incorrect males + incorrect females)]. All estimates were reported together with their corresponding 95% confidence intervals (95% CI), calculated using the exact Clopper–Pearson binomial method. The results are summarized in Table 2.

### 3.5. Ultrasound Evaluation

**Figure 8:**
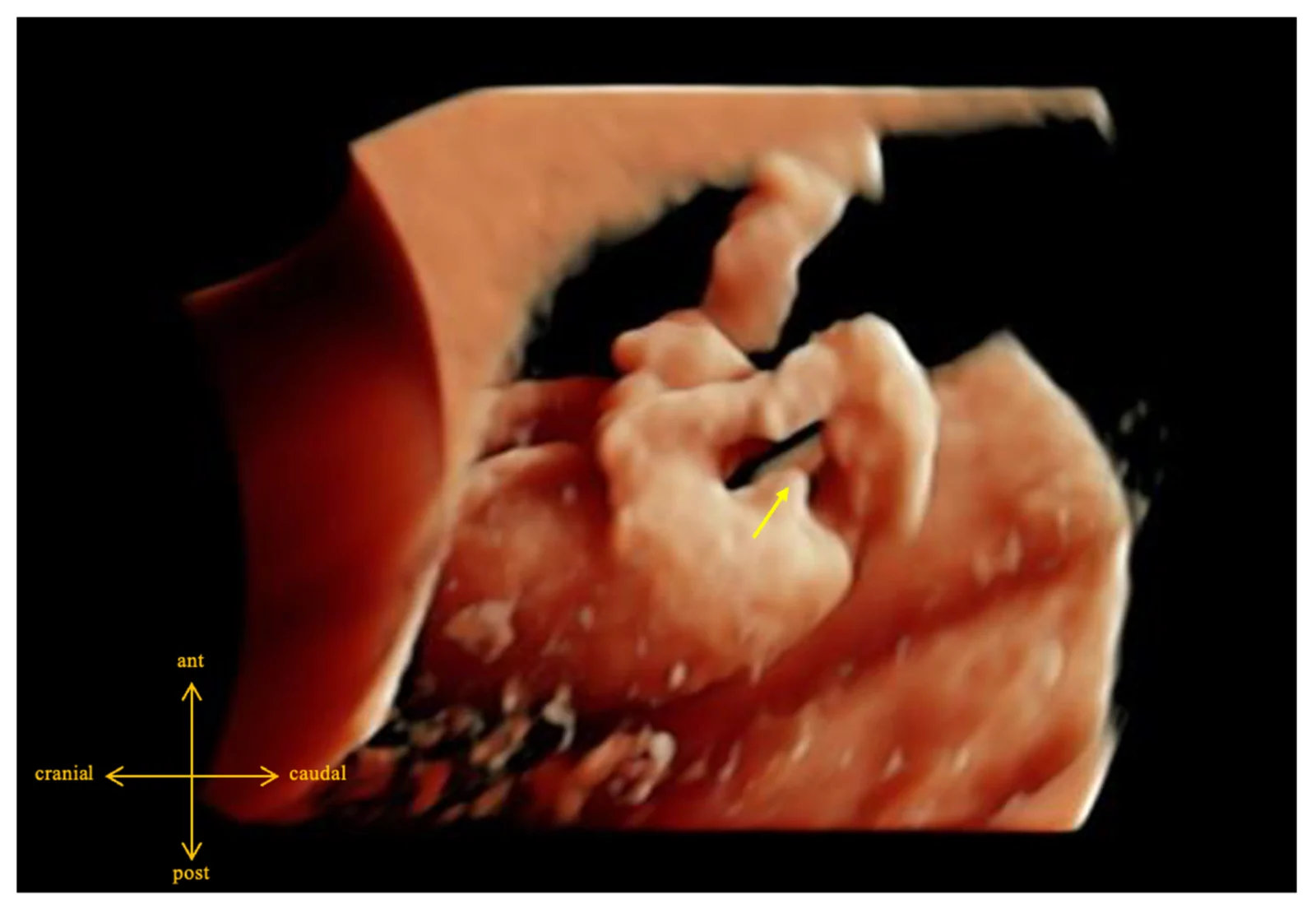
Ultrasonographic aspect of 8-week male individual. Note the morphology of the genital tubercle, along with its cranial direction, as it is seen from a sagittal plane (yellow arrow).

The ultrasound aspect in a male embryo at 8 weeks of gestation is consistent with the morphological aspect, illustrating the cranial orientation of the genital tubercle as seen in male embryos (see Figure 8).

**Figure 9:**
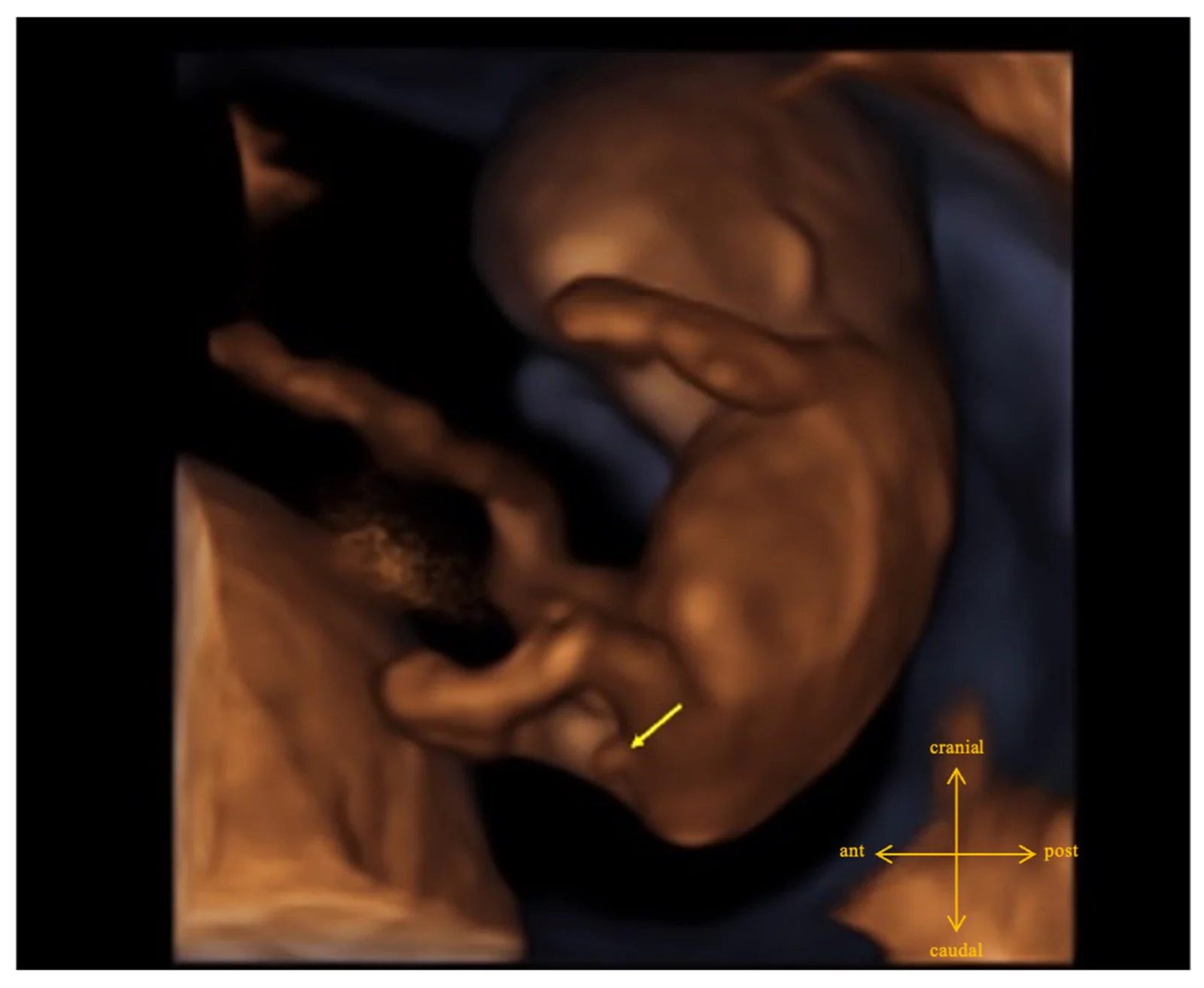
Ultrasonographic evaluation of the genital tubercle in an 8-week female fetus—the genital tubercle (yellow arrow) presents a caudal orientation, suggesting female sex.

Ultrasonographic examination of an 8-week female fetus illustrates the genital tubercle pointing towards inferior suggesting female sex, regardless of its dimensions (see Figure 9). Thus, fetal morphological studies show qualitative similarity between the macroscopic features and the ultrasound findings.

**Figure 10:**
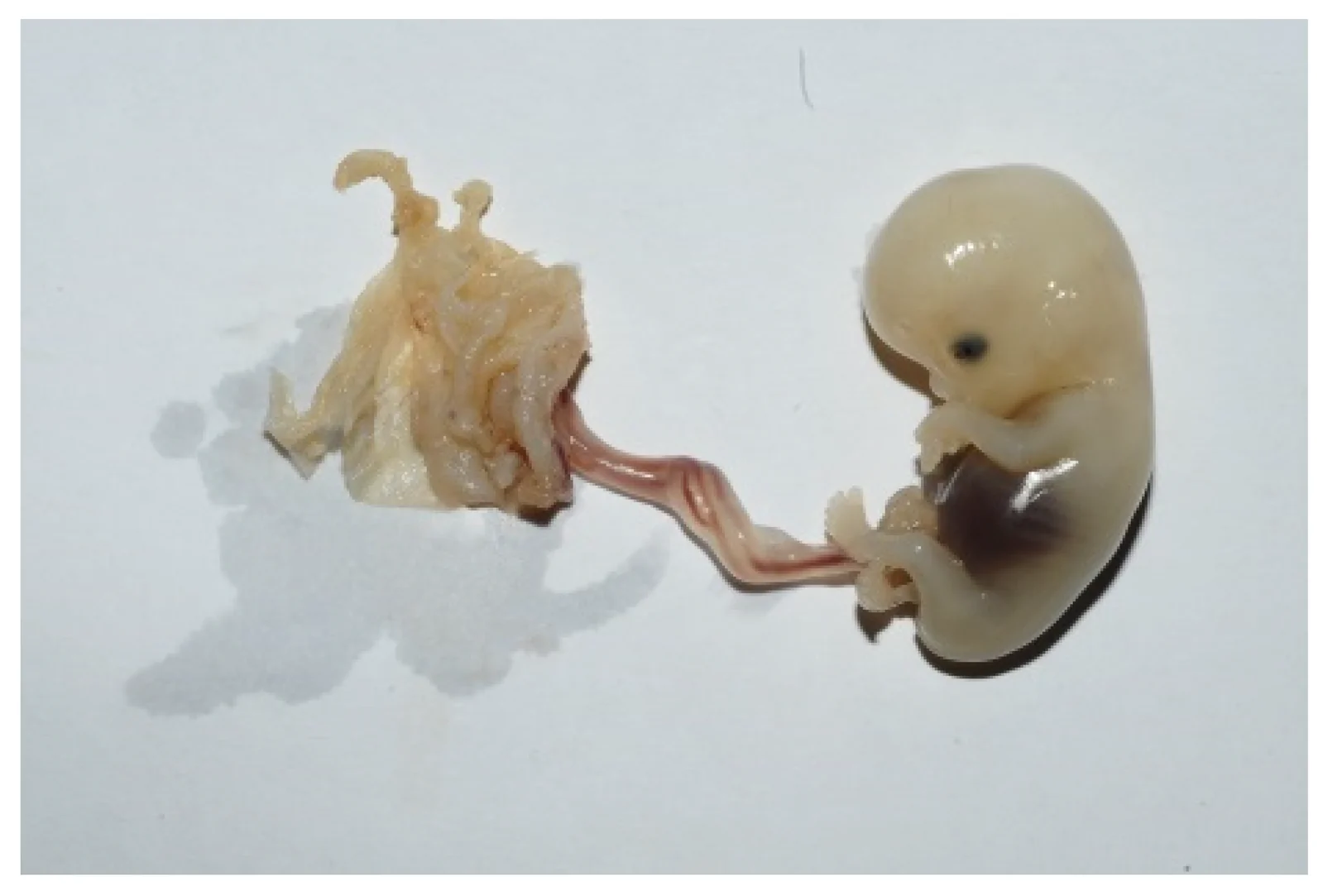
Lateral view of a 6–7-week fetus. Development of the lower limbs along with the position of the umbilical cord hinders the approach of the genital tubercle in both inspection of an ex vivo specimen and ultrasound assessment.

**Figure 11:**
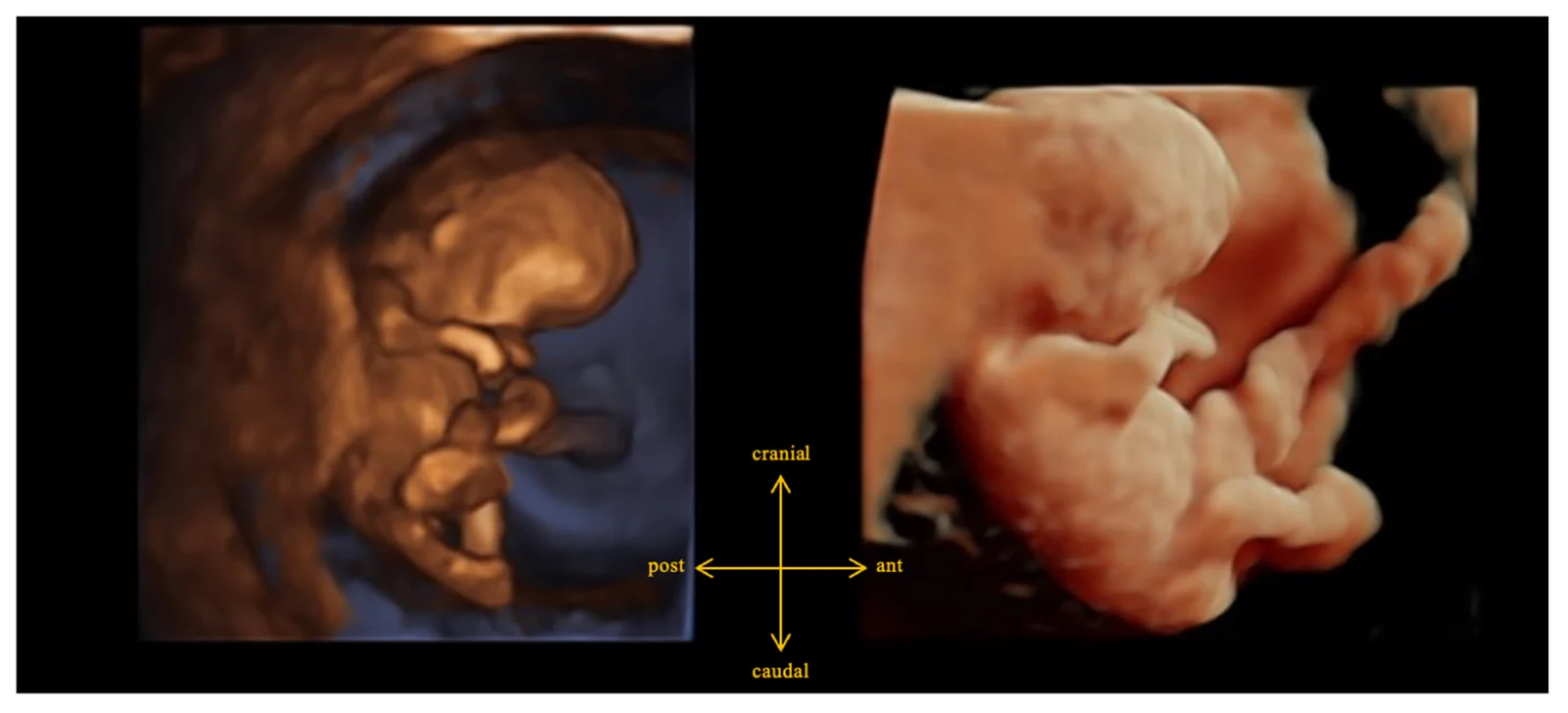
Ultrasonographic aspects of 10 weeks of gestation individuals—development of the lower limbs along with the position of the umbilical cord hinders the approach of the genital tubercle during ultrasound assessment.

Thus, we noticed that we are able to make an ultrasonographic determination of both the morphology of the genital tubercle and also its direction. However, in the process of determining fetal sex, there are impediments given both by the flexed position of the fetal lower limbs and by the presence of the umbilical cord (see Figure 10). Hence, the genital tubercle can be viewed with difficulty in certain situations. As shown in Figure 11, sex cannot be identified if the morphology and, more importantly, the direction of the genital tubercle is not visualized. An extensive ultrasound examination of the genial tubercle was performed in 150 cases. However, the examiners could establish the sex of the embryo in only 119 cases.

### 3.6. Statistical Analysis in the Ultrasonographic Branch of the Study

The 119 embryos and fetuses included in the ultrasonographic branch of the study have been categorized in three groups, depending on the gestational age of the specimens, as follows: 5 to 8 weeks of gestation (*n* = 24, 15 males and 9 females); 9 to 11 weeks of gestation (*n* = 43, 19 males and 24 females); 12 to 14 weeks of gestation (*n* = 52, 29 males and 23 females). The distribution of the embryos and fetuses and assessment of sex are summarized in Table 3. Diagnostic performance was further evaluated in a manner consistent with the procedures described in Section 3.4. Male sex was considered the positive outcome. Performance for fetal sex classification varied by gestational age and improved overall as gestational age increased. In the 5–8 week group (*n* = 24), overall accuracy was 54.17% (95% CI: 32.82–74.45%), with sensitivity for detecting males of 60.00% (95% CI: 32.29–83.66%) but low specificity of 44.44% (95% CI: 13.70–78.80%), indicating a considerable number of false-positive male classifications. In the 9–11 week group (*n* = 43), accuracy rose to 69.77% (95% CI: 53.87–82.82%), with sensitivity of 78.95% (95% CI: 54.43–93.95%) and higher specificity of 62.50% (95% CI: 40.59–81.20%). By 12–14 weeks (*n* = 52), accuracy reached 86.54% (95% CI: 74.21–94.41%), with sensitivity of 89.66% (95% CI: 72.65–97.81%) and specificity of 82.61% (95% CI: 61.22–95.05%). The results are summarized in Table 4.

## 4. Discussion

### 4.1. Morphological Studies Regarding the Genital Tubercle

To our knowledge, recent comprehensive studies covering the morphology of the fetal external genitalia and development of the genital tubercle are scarce in number. In two recent studies, Hulsman et al. [9] generated 3D models of the perineal region of several female human embryos and fetuses between 6 and 20 weeks, and analyzed the perineal skin in relation to the development of the external genitalia. Vieiralves et al. [10] conducted a morphological assessment of the variation of the anogenital distance (AGD) in female fetuses between 12 and 22 weeks, part of which were anencephalic. Several histological studies analyzed the developing female urethra and vagina, as well as the fetal penis [11].

One of the specimens included in our study presented an enlarged genital tubercle, suggestive of the male sex. Nevertheless, the genital tubercle pointed caudally, separating the two labio-scrotal swellings (see Figure 6). This case deserves more prominent discussion because it directly reinforces the central message of our study: in female fetuses, the orientation of the genital tubercle is more reliable than its apparent size when assigning sex. In this particular case, the seemingly enlarged genital tubercle should not be interpreted as true clitoromegaly. Rather, we believe its prominent appearance is explained by the relative paucity of the prepubic soft tissue that later contributes to the mons pubis (mons Veneris) and by the still-limited coverage provided by the developing labia majora. Developmental studies of female external genitalia show that, as gestation advances, the labia majora progressively cover the clitoris and the mons pubis becomes increasingly prominent, both of which reduce the visible prominence of the clitoral region [4,12]. Thus, an apparently larger tubercle in early or mid-gestation may reflect surrounding soft tissue anatomy rather than true enlargement of the clitoral structure itself [13].

This distinction is clinically relevant because true clitoromegaly implies actual enlargement of clitoral tissue and is classically associated with virilization or androgen excess, whereas a prominent clitoral/tubercle appearance may be transient and physiologic. Indeed, prior sonographic studies have documented chromosomally normal female fetuses with transient apparent clitoromegaly that resolved later in gestation and who had normal female genitalia at birth [14,15]. Likewise, the pediatric endocrine literature emphasizes that apparent clitoromegaly may result from insufficient surrounding soft tissue coverage rather than intrinsic clitoral hypertrophy [16]. This case is noteworthy and offers a valuable foundation for discussion; however, further investigation is required before any firm conclusions can be drawn. As a result, the findings cannot be reliably generalized, and definitive inferences regarding prevalence, causation, or clinical significance remain unestablished.

From a morphological perspective, assigning the sex of the embryo is possible in the bi-potential stage, with accuracy rates of 79.31% overall (81.25% in males and lower, of 76.92% in females) in our study, by assessing the orientation of the genital tubercle. However, from a clinical perspective, the ultrasonographic examination demonstrates lower accuracy rates in this gestational age group, of only 54.16% (60.00% in males, and 44.45% in females.

### 4.2. Determination of Fetal Sex by Ultrasound

In the late first and early second trimesters, fetal sex can be predicted by assessment of the direction in which the genital tubercle points—cranial for males and caudal for females—and also by the sagittal sign, whereby examination of the genital region in the midline sagittal plane demonstrates a caudal notch in females and a cranial notch in males [17]. The angle of the genital tubercle to a horizontal line through the lumbosacral skin surface can be measured. Consequently, the fetus is assigned male sex if the angle is >30°, and female sex if the genital tubercle is parallel or convergent (<10°) to the horizontal line [7]. At an intermediate angle of 10–30° the sex is not determined.

Prenatal determination of fetal sex by ultrasound during the second and third trimesters of pregnancy is based on the demonstration of and the size of the penis in males, or labial folds in females. There is no appreciable difference in the size of the penis and the clitoris until after 14 weeks of gestation. Mazza et al., in two studies [18,19], aimed to establish the accuracy of fetal sex assignment by sonography related to biparietal diameter (BPD). In the larger study of 2593 patients, at a BPD of 22 mm or greater, the accuracy rate was 99–100%.

Sex assignments now can be made as early as 13 weeks. During weeks 14 to 20, sex can be identified via contour by a midline sagittal view of the caudal end. The penis and scrotum are easiest to recognize, making a positive diagnosis for a male. Female sex should be assigned upon identification of the major and minor labia and by a sagittal view of the lower abdomen showing both the umbilical cord insertion and the caudally directed clitoris [20].

In recent decades, three small studies (Reece et al., 115 fetuses recruited [21]; Watson, 100 recruited [22]; Emerson et al., 165 recruited [23]) addressed high-resolution ultrasound in its ability to ascertain fetal sex. Meagher and Davison present a larger group of fetuses whose sex has been diagnosed in the early second trimester. Out of a recruited population of 843 fetuses, prenatal prediction was possible in 770 (91.3%) of the cases [24]. Along with the advancement of high-resolution ultrasound technology in the following 10 years, visualization rates have bettered greatly to 97–100%, although accuracy rates rest quite variably (92–100%) [25].

Transvaginal ultrasonography enables sex determination at an early stage of pregnancy. The main diagnostic criteria for male sex determination are the “dome” sign representing the visualization of the fetal scrotum, the cranially directed phallus, and the longitudinal raphe at the base of the penis. The diagnostic criteria for female sex are the two or four parallel lines and the caudally directed phallus [26].

Our study focused on early determination of fetal sex in the first trimester. We were able to correctly assign the fetal sex by ultrasound examination in only 54.16% (*n* = 13) of the embryos between 5 and 8 weeks of gestation. Between 9 and 11 weeks of gestation, we correctly assigned the sex of the fetus in 69.76% (*n* = 30) of the cases. Similarly, Hsiao et al. accurately assigned the sex in 71.93% (*n* = 41) of the fetuses of 11 weeks of gestation [27] and Efrat et al. correctly assigned the sex in 70.3% fetuses of 11 weeks of gestation [17]. In comparison, Gharekhanloo et al. correctly assigned the sex, when attempted, in 91.3% of the fetuses of 11 weeks of gestation, with an overall accuracy rate of 45% at this gestational age [7].

Between 12 and 14 weeks of gestation, we correctly assigned the sex of the fetus in 86.53% (*n* = 45) of the cases with a higher accuracy in males (89.65%) than females (82.60%). Hsiao et al. demonstrated a total accuracy rate of 87.05% (*n* = 195), with a 73.30% accuracy in males and 70.30% in females [27]. Gharekhanloo et al. correctly assigned the sex, when attempted, in 90.5% of the fetuses of 11 weeks of gestation, with an overall accuracy rate of 67.7% at this gestational age [7]. In comparison, Efrat et al. correctly identified the gender in 98.7% of the fetuses in the 12th week of gestation, and 100% of the cases at 13 weeks [17].

A more recent approach in determining the fetal sex through ultrasound implies measuring the distance between the anus and the base of the genital tubercle, known as the AGD. Several studies concluded that AGD can establish sex as early as the first trimester with good reproducibility. Although fetal genitalia measurements may be useful in predicting sex at advanced gestational ages, they are not very useful in early pregnancy [28].

3DUS has been proven in many studies to be superior to 2DUS in revealing fetal abnormalities [29,30]. Past studies have shown that sex can be assigned with 3DUS, especially because of the ease of rotating the volume to the preferred midsagittal plane [31]. However, reproducibility is not a general property of the ultrasound examination; it is parameter-specific, so even when measurements come from the same examination, different variables can show markedly different agreement. In particular, studies such as the recent FetalHQ [32] work suggest that some geometric/basic structural parameters can achieve higher reproducibility, while several functional measures—including stroke volume, cardiac output, and global longitudinal strain—often display only moderate to lower reproducibility, limiting how confidently they can be compared across observers or repeated acquisitions.

Identification of fetal sex can also be performed on MRI from 18 weeks, as MRI may help to better visualize different anomalies of the fetal external genitalia in comparison to prenatal ultrasound [33].

### 4.3. Limitations of the Study

A key limitation of this study is that the two cohorts were derived from fundamentally different populations. Specifically, the anatomical specimens were obtained from aborted fetuses, whereas the ultrasound measurements were collected from ongoing pregnancies. Because fetal development, maternal and environmental conditions, and potential viability-related factors may differ between these settings, the groups may not be directly comparable. As a result, this discrepancy could introduce selection bias and influence the observed rates and characteristics of the findings, which in turn may limit the generalizability of the results to broader clinical contexts.

Secondly, we acknowledge as a limitation that genetic confirmation of fetal sex was performed in only a small subset of specimens. For most cases, sex determination was based on inspection of internal genital anatomy at necropsy. Although this approach is typically highly reliable in advanced specimens, it remains a secondary standard compared with genetic verification. Therefore, this methodological difference could introduce misclassification risk in any cases where internal genital features are less definitive or overlap due to developmental variation.

Moreover, we were unable to evaluate the sex in 31 of the subjects. At earlier gestational ages, visualization is more sensitive to both fetal position and umbilical cord interference because the fetus and cord occupy a smaller field within the uterus and often lie at more variable angles relative to the ultrasound beam. Small changes in alignment can therefore prevent the target anatomy from being optimally insolated, producing reduced acoustic coupling and weaker image contrast. In addition, because the gestational tissues are still developing and are less distinct structurally, any motion, partial occlusion, or shadowing caused by the cord can disproportionately obscure critical features, leading to lower rates of clear identification compared with later gestations, when visualization can still be affected, but the effects are often more predictable and, in many cases, less disproportionate. The fetus is larger and tends to occupy a greater, more consistent portion of the imaging window, making key structures easier to align with the ultrasound beam [34,35].

Although the anatomical findings are comprehensive and informative, routine clinical applicability of fetal sex determination through ultrasound examination at 6–8 weeks is likely limited at this point. The ability to discern relevant structures under controlled macroscopic laboratory conditions does not directly equate to what can be reliably achieved through routine first-trimester ultrasound in everyday obstetric practice. Nonetheless, extensive future work is needed, including multicenter prospective validation to confirm reproducibility and generalizability, standardized 3D volume acquisition protocols to reduce technical variability, and the potential use of AI-assisted image segmentation to enhance detection accuracy and streamline workflow. Recent studies have already determined the potential use of AI to automatically identify standardized fetal head imaging planes and support more consistent fetal skull biometry while reducing the time required for ultrasound assessment [36]. Collectively, these steps should strengthen the evidence base for clinical feasibility and support translation from anatomical observation toward a more robust and scalable implementation.

## 5. Conclusions

The orientation of the genital tubercle plays an essential role in establishing the sex of the fetus, regardless of its size. The genital tubercle orientates cranially in males and caudally in females as early as 6 weeks of gestation, during the bi-potential phase. By analyzing the direction of the genital tubercle, the examiner may be able to determine the gender of the fetus early in the pregnancy. However, rather than enabling definitive sex determination at 5–6 weeks, our macroscopic findings demonstrate that subtle directional orientation differences in the genital tubercle may be discernible prior to complete phenotypic divergence. As a precaution, the sex of the fetus should be reconfirmed later in the course of the pregnancy, preferably at up to 20 weeks’ gestation.

## Figures and Tables

**Table 1:** Distribution by sex and the assessment of embryological and fetal sex by morphological analysis between 5 and 14 weeks of gestation.

Gestational Age (Weeks)	5–8 Weeks No. (%)	9–11 Weeks No. (%)	12–14 Weeks No. (%)
Correct Male	13 (81.25%)	17 (89.47%)	36 (100.00%)
Incorrect Male	3 (18.75%)	2 (10.53%)	0 (0.00%)
Correct Female	10 (76.92%)	22 (88.00%)	32 (100.00%)
Incorrect Female	3 (23.08%)	3 (12.00%)	0 (0.00%)
Total Correct	23 (79.31%)	39 (88.64%)	68 (100.00%)
Total Incorrect	6 (20.69%)	5 (11.36%)	0 (0.00%)
Total	29 (100.00%)	44 (100.00%)	68 (100.00%)

**Table 2:** Gestational-age-dependent accuracy and predictive values in sex determination by morphological analysis. CI—confidence interval.

	5–8 Weeks	9–11 Weeks	12–14 Weeks
Sensitivity	81.25% (95% CI: 61.7–94.4)	89.47% (95% CI: 74.1–97.5)	100.00% (95% CI: 89.6–100)
Specificity	76.92% (95% CI: 56.2–90.1)	91.67% (95% CI: 73.7–98.9)	100.00% (95% CI: 89.1–100)
PPV	81.25% (95% CI: 56.0–94.9)	89.47% (95% CI: 66.6–97.6)	100.00% (95% CI: 90.2–100)
NPV	76.92% (95% CI: 50.7–93.0)	91.67% (95% CI: 70.7–99.0)	100.00% (95% CI: 89.1–100)
Accuracy	79.31% (95% CI: 63.5–90.2)	88.63% (95% CI: 75.6–95.2)	100.00% (95% CI: 94.6–100.0)

**Table 3:** Distribution by sex and the assessment of embryological and fetal sex by ultrasound between 5 and 14 weeks of gestation.

Gestational Age (Weeks)	5–8 Weeks, No. (%)	9–11 Weeks, No. (%)	12–14 Weeks, No. (%)
Male correctly classified (true positive)	9 (60.00%)	15 (78.95%)	26 (89.66%)
Male incorrectly classified as female (false negative)	6 (40.00%)	4 (21.05%)	3 (10.34%)
Female correctly classified (true negative)	4 (44.44%)	15 (62.50%)	19 (82.61%)
Female incorrectly classified as male (false positive)	5 (55.56%)	9 (37.50%)	4 (17.39%)
Total Correct	13 (54.17%)	30 (69.77%)	45 (86.54%)
Total Incorrect	11 (45.83%)	13 (30.23%)	7 (13.46%)
Total	24 (100.00%)	43 (100.00%)	52 (100.00%)

**Table 4:** Gestational-age-dependent diagnostic performance of fetal sex classification by ultrasonographic evaluation. Male sex was considered the positive outcome. PPV, positive predictive value; NPV, negative predictive value; LR+, positive likelihood ratio; LR−, negative likelihood ratio; CI, confidence interval. Exact 95% confidence intervals for sensitivity, specificity, PPV, NPV, and accuracy were calculated using the Clopper–Pearson method; confidence intervals for likelihood ratios were calculated using the logarithmic method.

	5–8 Weeks	9–11 Weeks	12–14 Weeks
Sensitivity	60.00% (95% CI: 32.29–83.66)	78.95% (95% CI: 54.43–93.95)	89.66% (95% CI: 72.65–97.81)
Specificity	44.44% (95% CI: 13.70–78.80)	62.50% (95% CI: 40.59–81.20)	82.61% (95% CI: 61.22–95.05)
PPV	64.29% (95% CI: 35.14–87.24)	62.50% (95% CI: 40.59–81.20)	86.67% (95% CI: 69.28–96.24)
NPV	40.00% (95% CI: 12.16–73.76)	78.95% (95% CI: 54.43–93.95)	86.36% (95% CI: 65.09–97.09)
LR+	1.08 (95% CI: 0.53–2.21)	2.11 (95% CI: 1.20–3.71)	5.16 (95% CI: 2.10–12.67)
LR−	0.90 (95% CI: 0.35–2.35)	0.34 (95% CI: 0.13–0.85)	0.13 (95% CI: 0.04–0.37)
Accuracy	54.17% (95% CI: 32.82–74.45)	69.77% (95% CI: 53.87–82.82)	86.54% (95% CI: 74.21–94.41)

## Data Availability

The data presented in this study are available on request due to restrictions (legal and ethical reasons) from the corresponding author.
